# A CBL-Interacting Protein Kinase TaCIPK2 Confers Drought Tolerance in Transgenic Tobacco Plants through Regulating the Stomatal Movement

**DOI:** 10.1371/journal.pone.0167962

**Published:** 2016-12-09

**Authors:** Yan Wang, Tao Sun, Tingting Li, Meng Wang, Guangxiao Yang, Guangyuan He

**Affiliations:** The Genetic Engineering International Cooperation Base of Chinese Ministry of Science and Technology, The Key Laboratory of Molecular Biophysics of Chinese Ministry of Education, College of Life Science and Technology, Huazhong University of Science and Technology, Wuhan, China; Institute of Genetics and Developmental Biology Chinese Academy of Sciences, CHINA

## Abstract

In plants, the CBL-CIPK signaling pathways play key roles in the response to abiotic stresses. However, functional studies of CIPKs in the important staple crop wheat are very rare. In this study, we identified a *CIPK* gene from wheat, designated *TaCIPK2*. Expression analysis results showed that *TaCIPK2* could be up-regulated in wheat leaves by polyethylene glycol, abscisic acid and H_2_O_2_ treatments. Subcellular localization analyses revealed that TaCIPK2 was present in whole wheat epidermal cells. A yeast two-hybrid assay indicated that TaCIPK2 interacted with TaCBL1, 2, 3 and 4 in vitro. Transgenic tobacco plants over-expressing *TaCIPK2* exhibited increased drought tolerance, indicated by a larger proportion of green cotyledons and higher survival rates under the osmotic and drought stress conditions compared with control plants. Additionally, physiological index analyses revealed that the transgenic tobacco plants had lower water loss rates and ion leakage, accumulated less malondialdehyde and H_2_O_2_, and had higher catalase and superoxide dismutase activities than the control plants. The transgenic plants also exhibited faster stomatal closure following exposure to osmotic stress conditions. The seed germination rates and stomatal aperture of *TaCIPK2*-overexpressing tobacco plants decreased after exogenous abscisic acid treatment was applied, implying that the transgenic tobacco plants were more sensitive to exogenous abscisic acid than the control plants. Our results indicate that TaCIPK2 plays a positive regulatory role in drought stress responses in transgenic tobacco plants.

## Introduction

Calcium acts as a versatile signaling molecule during plant responses to external stimuli, including cold, salt and drought stress [1, 2]. Cellular Ca^2+^ signals are perceived and transferred by many sensors, including calcineurin B-like proteins (CBLs), which then transmit these signals to downstream functional proteins [3]. The CBL-interacting protein kinases (CIPKs) in plants decode the Ca^2+^ signals from CBLs [4, 5].

To date, *CIPK* family genes have been identified in Arabidopsis, rice, canola, maize and wheat, and some *CIPKs* have been functionally characterized [5–9]. The AtCBL4-CIPK24 (SOS3-SOS2) complex specifically regulates Na^+^ efflux by the Na^+^/H^+^ antiporter and enhances salt tolerance [10, 11]. The overexpression of many *CIPK* genes increases salt tolerance in plants, including *ZmCIPK21* in maize [12], *HbCIPK2* in *Hordeum brevisubulatum* [13], *PeCIPK24-26* in *Populus* species [14], and *TaCIPK14* in wheat [15]. These genes are involved in the salt overly sensitive (SOS) signaling pathway. In addition to participating in regulation of Na^+^, CIPKs are involved in regulating other ions. Several CIPKs have recently been reported to be involved in regulating the K^+^ transporter. In Arabidopsis, a voltage-gated inward K^+^ channel (AKT1) is phosphorylated by AtCIPK23, leading to increased K^+^ uptake under low-potassium conditions [16]. AtCIPK23/AtCIPK9 activates AKT1 by phosphorylation at the cell membrane [16, 17]. The *akt1/cipk23* Arabidopsis mutant exhibits more efficient stomatal closure and greater sensitivity to drought stress because of an altered inward-rectifying K^+^ current [18]. In rice, OsCIPK23, which is a homolog of AtCIPK23, activates the Os-AKT1 channel in oocytes. The RNAi-mediated silencing of *Os-CIPK23* in rice plants resulted in a potassium-deficient phenotype, similar to that of the *Osakt1* mutant under low-potassium stress condition [19]. Knockout mutants and complementation analyses indicated that AtCIPK8 helps to regulate the low-affinity nitrate responses [20]. A series of mutant analyses revealed that Arabidopsis CBL2/3 and CIPK3/9/23/26 combine to form multiple interacting networks that protect plants from Mg^2+^ toxicity by regulating the vacuolar sequestration of Mg^2+^ [21]. Moreover, the AtCBL1/AtCBL9 and AtCBL2/AtCBL3 complexes regulate Arabidopsis pollen tube growth and affect embryonic morphology respectively, suggesting that CBL/CIPK networks play multiple roles during plant development [22, 23].

Although considerable progress has been made in characterizing CIPKs in many plants, only a few of them have been functionally analyzed in the staple crop wheat, because of its hexaploid nature. In this study, we found that the *TaCIPK2*, a *TaCIPK* gene from wheat, was responsive to multiple abiotic stresses. The over-expression of *TaCIPK2* confers drought tolerance in transgenic tobacco plants, at least in part by regulating stomatal closure.

## Materials and Methods

### Sequence analysis of *TaCIPK2*

Full-length *TaCIPK2* (GenBank: KJ561791.1) cDNA was cloned from wheat (*Triticum aestivum* L. cv. Chinese spring) in our previous study [7]. The other CIPK2 proteins of *Triticum urartu*, *Aegilops tauschii*, *Hordeum brevisubulatum*, *Hordeum vulgare*, *Brachypodium distachyon*, *Arabidopsis thaliana* and *Oryza sativa* were obtained from NCBI (https://blast.ncbi.nlm.nih.gov/Blast.cgi) and the comparative analysis of the above-mentioned sequences was performed using the ClustalX2.0 software. The phylogenetic tree of CIPK2 proteins has been constructed in the MEGA 5.0 software. The predicted myristoylation and palmitoylation sites of TaCIPK2 were analysis using the GPS-Lipid 1.0 software.

#### Plant materials and stress treatments

Sterilized wheat (*Triticum aestivum* L. cv. Chinese spring) seeds were germinated under a 10-h light/14-h dark cycle at 22°C in a phytotron. Two-week-old seedlings from the hydroponic culture (endosperm could still provide necessary nutrient) were watered and sprayed with 200 mM NaCl (Sinopharm, China), 20% polyethylene glycol (PEG) 6000 (Biosharp, China), 10 mM H_2_O_2_ (Sinopharm, China), or 100 μM abscisic acid (ABA, Biosharp, China). The untreated and treated of wheat leaves were sampled at 0, 1, 3, 6, 9, 12, and 24 h at the same time. To analyze expression patterns, the flag leaves, pistils and stamens as well as seedling and flowering stage roots, stems and leaves were harvested and stored at -80°C.

### Subcellular localization of TaCIPK2

To determine the subcellular localization, the coding region of TaCIPK2 was cloned using primers containing *Bma*H I/*Sma* I restriction sites and fused into the GFP-containing pBI121 expression vector under the cauliflower mosaic virus 35S promoter. The primers containing the *Bma*H I/*Sma* I restriction sites are listed in S1 Table. *Agrobacterium tumefaciens* EHA105 cells were transformed with the pBI121-*TaCIPK2-GFP* and pBI121-*GFP* plasmids. Fresh *A*. *tumefaciens* cultures were resuspended in medium containing 10 mM 2-(N-morpholino) ethanesulfonic acid (MES), 10 mM MgCl_2_, and 10 μM acetosyringone and then infiltrated into wheat seeding leaves. Four or five days later, green fluorescence signals were observed using fluorescence microscopy (LX71, Olympus, Japan).

To make sure the TaCIPK2 was located on the plasma membrane, the pBI121-*TaCIPK2-GFP* vector packed by gold power was bombarded into onion (*Allium cepa* L.) epidermal cells by particle bombardment (PDS-1000, Bio-Rad, USA). After incubating in room temperature for 24 h, the tissue was treated with 30% sucrose solution for 20 minutes. The green fluorescence signals were photoed by fluorescence microscopy (LX71, Olympus, Japan).

### Yeast two-hybrid assay

The MatchMaker yeast two-hybrid system was used for yeast two-hybrid analysis (Clontech, USA). The coding regions of the *TaCBL* genes (*TaCBL1–4*, *TaCBL6*, *TaCBL7*, and *TaCBL9*) and *TaCIPK2* were inserted into the pGBKT7 and pGADT7 vectors, respectively. The pGBKT7-*TaCBLs* and pGADT7-*TaCIPK2* plasmids along with the positive and negative controls were co-transformed into the AH109 yeast strain. The transformants were first grown on double-dropout medium (SD/-Trp/-Leu), and after three days, the growth transformants were transferred to triple-dropout medium (SD/-Trp/-Leu/-Ade) and quadruple-dropout medium (SD/-Ade/-His/-Trp/-Leu) with or without X-α-gal. Photographs were taken after 5 days.

### Stomatal closure and density assay

The stomatal closure assay was performed using leaves harvested from 30-day-old control or transgenic tobacco seedlings as described previously [24]. The leaves were immersed in a solution containing 10 mM KCl, 50μM CaCl_2_, and 10 mM MES-KOH (pH 6.15) under strong light for 6 h. Subsequently, 50 μM ABA or 200 mM mannitol was added to the solution. The stomata status was assessed by microscopy (LX71, Olympus, Japan) 3 or 1 h after 50 μM ABA or 200 mM mannitol treatment, respectively. In addition, a density assay was performed as previously described [25].

### Stress tolerance assay using transgenic tobacco plants

The coding region of TaCIPK2 was cloned using primers containing *Bma*H I/*Sma* I restriction sites and inserted into the pBI121 expression vector under the cauliflower mosaic virus 35S promoter. Tobacco plants (*Nicotiana tabacum* L. cv Samsun) were transformed with the vacant vector pBI121 (VC) or the recombinant plasmid pBI121-*TaCIPK2* using an *Agrobacterium*-mediated leaf disc transformation method [26]. Three independent transgenic (OE1, OE2 and OE4) and VC tobacco lines were germinated on selective medium containing 100 mg/L of kanamycin. The gene expression levels in transgenic tobacco plants were measured using reverse transcription polymerase chain reaction (RT-PCR) method.

The T_3_ generations of three independent *TaCIPK2*-overexpressing lines along with the wild-type (WT) and VC controls were used for drought stress tolerance assay. To determine the effects of mannitol and ABA treatments on seed germination, seeds from the controls and transgenic plants were grown on basal Murashige and Skoog (MS) medium and MS media containing 200 or 300 mM mannitol and 0.5 μM ABA. Seed germination rates (the proportion of green cotyledons) were determined after 2 weeks. To assess the effects of drought stress, 5-week-old tobacco seedlings were deprived of water for 3 weeks, then watered regularly for 1 week. The number of surviving plants was then counted. All experiments were repeated three times.

### Measurement of physiological indices

To detect changes in physiological indices under normal and drought stress conditions, leaves were collected from the controls and *TaCIPK2*-overexpressing transgenic lines and treated with 0.1 M phosphate-buffered saline (pH 7.4) on ice. The crude extract was centrifuged at 8,000 g for 10 min at 4°C and the supernatants were used to measure the physiological index changes. The malondialdehyde (MDA) and H_2_O_2_ contents and the antioxidant enzyme activities [catalase (CAT), superoxide dismutase (SOD), and peroxidase (POD)] were measured using the corresponding detection kits (A003-3, A064, A007-1, A001, and A084-3; Jiancheng, China). Ion leakage (IL) was measured under normal and drought stress conditions. Leaves of all lines were harvested and incubated in 15 ml distilled water overnight at 23°C before measuring the initial conductivity (C1). After boiling the samples for 30 min, the final conductivity (C2) was determined. The following formula was used to calculate IL (%): C1/C2×100.

### Real-time quantitative PCR (qRT-PCR) analysis

Quantitative RT-PCR (qRT-PCR) was used to determine the *TaCIPK2* expression patterns in various wheat organs and the leaves and roots of seedlings treated with various stresses including NaCl, PEG 6000, ABA, and H_2_O_2_. Total RNA extracted from samples using the Plant Total RNA Extraction Kit (Zomanbio, China) served as the template for cDNA synthesis using the FastQuant RT kit (Tiangen, China). Gene-specific primer pairs were designed according to the non-conserved domain and the 3’-untranslated region sequences (S1 Table). The relative gene expression levels according to the qRT-PCR data were calculated using the 2^−ΔΔCT^ method [27].

### Statistical analysis

All the data were analyzed in Excel, and the three independent experiments were used to calculate the mean values ± SD. A one-way analysis of variance was applied to identify statistically significant differences.

## Results

### Analysis of *TaCIPK2* from *T*. *aestivum*

**Fig 1:**
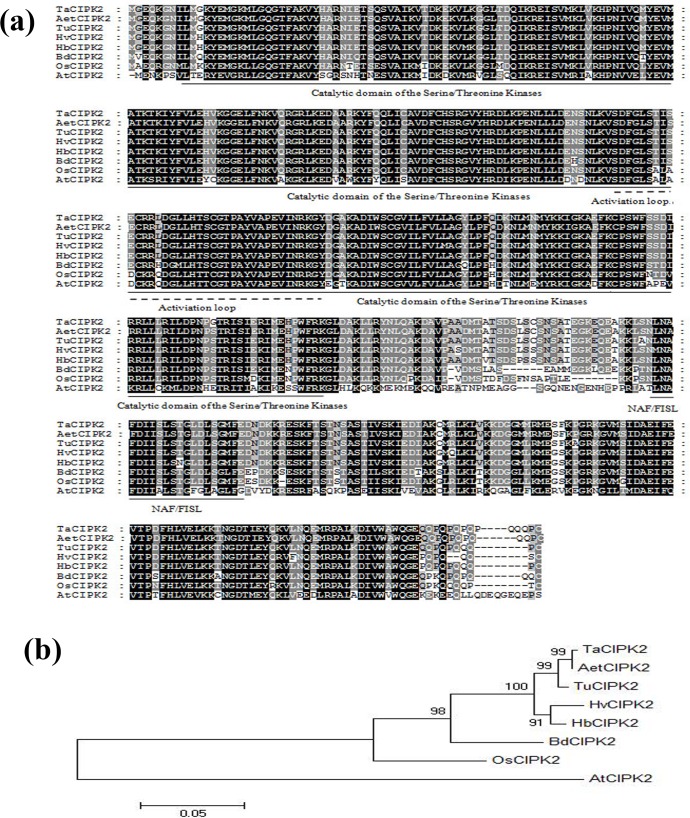
Characterization and sequence analysis of TaCIPK2. (a) Alignment of TaCIPK2protein with related CIPKs from other plant species. (b) Phylogenetic analysis of CIPK2 proteins in several plants.

The coding region (CDS) of TaCIPK2 was 1,368 bp and encoded 456 amino acids. The TaCIPK2 protein had a serine/threonine kinase catalytic domain, an activation loop and a NAF/FISL domain, which were similar to other CIPK2 proteins. BLAST was used to analyze the homology between TaCIPK2 and other CIPK2 proteins. The results showed that the similarities between TaCIPK2 and AetCIPK2 (EMT16422.1), HvCIPK2 (AKL71570.1), HbCIPK2 (AET80728.1), BdCIPK2 (NP_00130481) and OsCIPK2 (XP_015646996.1) were 99%, 96%, 97%, 88% and 84%, respectively. In addition, the MEGA 5.0 software was used to perform multiple alignment and phylogenetic analyses, and the results showed that the similarity between TaCIPK2 and AetCIPK2 was above 90% suggesting the possibility that these proteins from different species may have originated from a common ancestor (Fig 1).

#### *TaCIPK2* expression is responsive to multiple abiotic stresses

**Fig 2:**
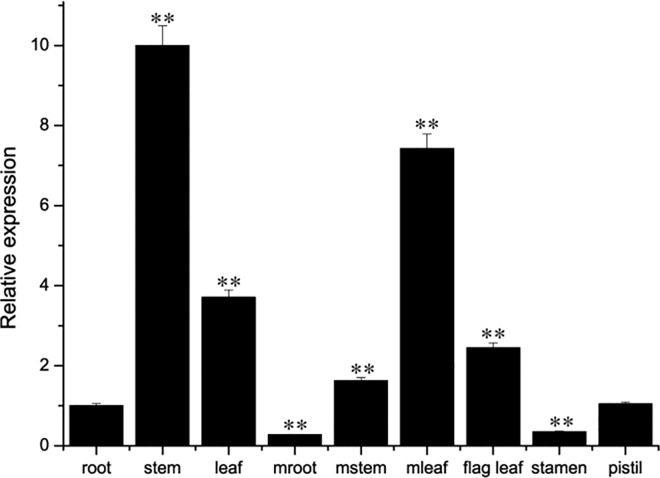
Organ expression analysis of *TaCIPK2* in wheat. Different wheat organs including the root, stem and leaf from young seedlings, and the root, stem leaf, flag leaf, and reproductive organs (stamen and pistil) from mature plants. The relative expression levels of *TaCIPK2* were analyzed by the 2^−ΔΔCT^ method. Significant differences between the root and other organs are indicated as *P < 0.*05*; **P < 0.*01*.

To assess *TaCIPK2* expression patterns, RNA was isolated from 11 wheat organs for qRT-PCR analyses. The results showed that *TaCIPK2* was expressed in all the organs examined at varying levels. Higher expression levels were observed in stems, young leaves and mature leaves, while lower expression levels occurred in mature roots and stamens (Fig 2).

**Fig 3:**
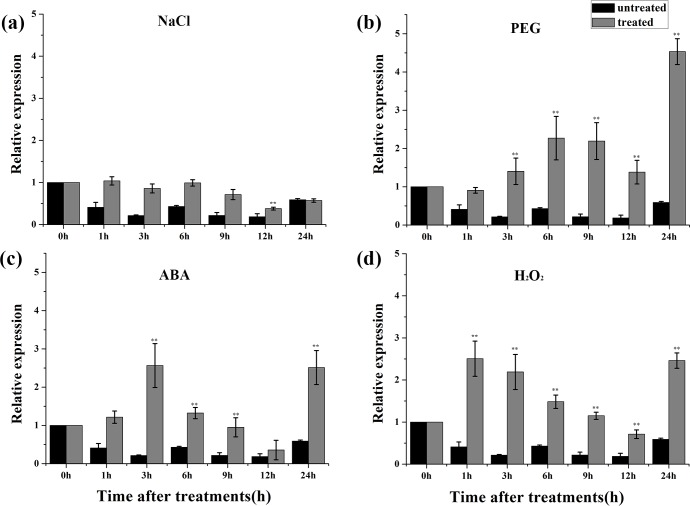
Expression patterns of *TaCIPK2* under normal conditions and stress treatments with NaCl, PEG, ABA, and H_2_O_2_ in wheat leaves by qRT-PCR analysis. The 2-week-old wheat leaves were treated with NaCl (a)., PEG 6000 (b), ABA (c) and H_2_O_2_ (d). The 2^−ΔΔCT^ method was used to analyze the relative expression of TaCIPK2. Asterisks indicate statistically significant differences (*P < 0.*05*; **P < 0.*01*) compared to untreated wheat at 0 h. Three independent experiments were performed and error bars show the SD.

To further examine whether *TaCIPK2* expression is affected by abiotic stresses and exogenous ABA, the leaves of 14-day-old wheat seedlings were harvested after treatment with or without NaCl, PEG 6000, H_2_O_2_, and ABA. Besides NaCl stress, the *TaCIPK2* transcript level was considerably increased by the PEG 6000, H_2_O_2_, and ABA treatments in leaves (Fig 3).These results suggested the *TaCIPK2* was responsive to PEG 6000, H_2_O_2_ and ABA, and may participate in the regulation of plant tolerance to these abiotic stress conditions.

### TaCIPK2 is localized in the nucleus, cytoplasm and plasma membrane of the wheat epidermal cell

**Fig 4:**
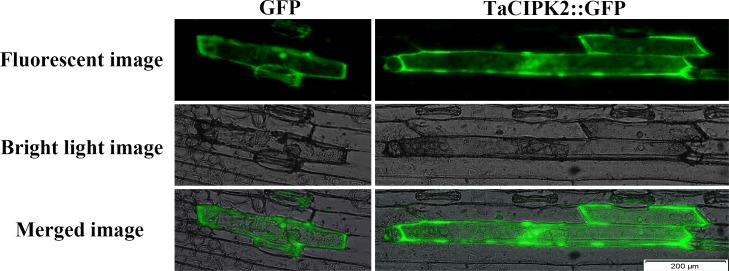
Subcellular localization of GFP and TaCIPK2::GFP fusion protein in wheat epidermal cells. The fusion protein CIPK2-GFP and GFP (control) were transiently expressed in wheat epidermal cells. Scale bar = 200 μm. The green fluorescence signals were determined by fluorescence microscopy (LX71, Olympus, Japan).

To clarify TaCIPK2 localization in wheat cells, a *TaCIPK2-GFP* fusion protein expression vector under the control of the *cauliflower mosaic virus* (CaMV) 35S promoter was constructed. The plasmids containing the 35S:*TaCIPK2-GFP* and 35S:*GFP* (control) sequences were infiltrated into wheat leaves using an *Agrobacterium*-mediated transformation method. The green fluorescence signal resulting from the expression of TaCIPK2-GFP was visible throughout the entire epidermal cell, similar to the fluorescence signal of GFP alone (Fig 4). In addition, the plasmolysis experiment of the onion epidermis cells showed that the TaCIPK2 was located on the nucleus, cytoplasm and plasma membrane (S1 Fig). The plasma membrane location of TaCIPK2 was consistent with the predicted two myristoylation sites and one palmitoylation site (S2 Fig). The localization of TaCIPK2 was consistent with the localization of other CIPK proteins, indicating that the TaCIPK2-GFP fusion protein was present and potentially functional throughout the wheat cell, including the nucleus, cytoplasm and plasma membrane.

### Interaction between TaCIPK2 and TaCBL proteins in a yeast two-hybrid assay

**Fig 5:**
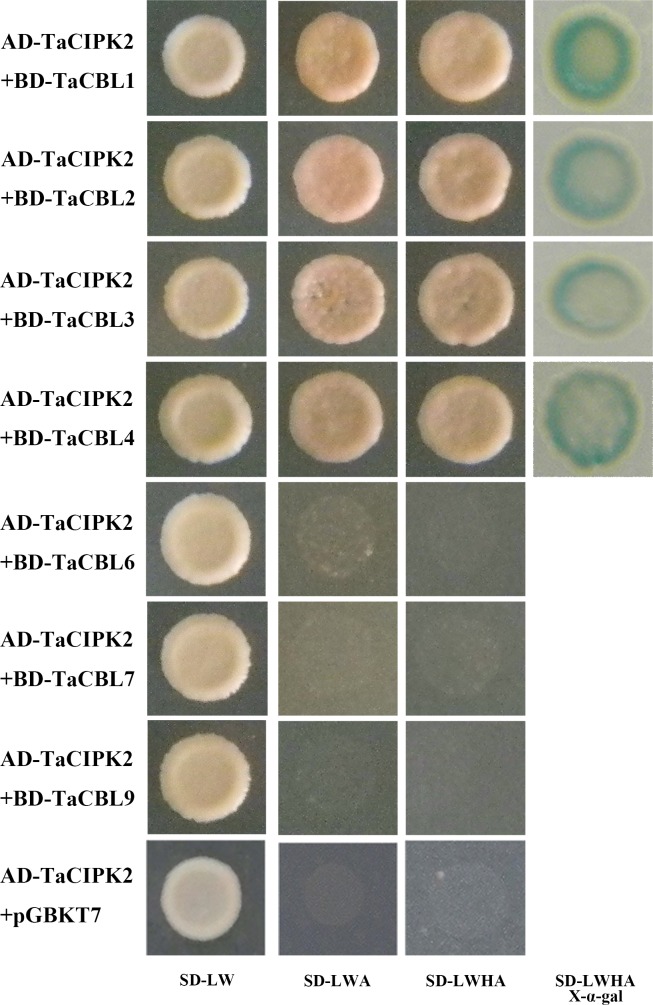
Yeast two-hybrid analysis of TaCIPK2-TaCBLs interaction. The pGBKT7-TaCBLs and pGADT7-TaCIPK2 plasmids were co-transformed into the AH109 yeast strain and the transformants were selected on SD/-Trp/-Leu, SD/-Trp/-Leu/-Ade and SD/-Ade/-His/-Trp/-Leu with or without X-α-gal.

The interactions between the CBL and CIPK proteins are very complex, with one CBL protein being able to interact with one or multiple CIPK proteins to fulfill different functions of CIPKs in plants. To identify the interaction partners of TaCIPK2, a yeast two-hybrid assay was used to examine the interactions between TaCBL proteins and TaCIPK2. Seven *TaCBL* genes were inserted into pGBKT7, and TaCIPK2 was cloned into pGADT7. The vectors were then used to transform cells of the yeast strain AH109, which were firstly grown on non-selective medium (SD-LW), then transferred to selective media (SD-LWA and SD-LWHA) to screen for positive interactions. The results showed that TaCIPK2 can interact with TaCBL1, 2, 3 and 4, at least in vitro (Fig 5).

### TaCIPK2 enhances drought tolerance of transgenic tobacco plants

**Fig 6:**
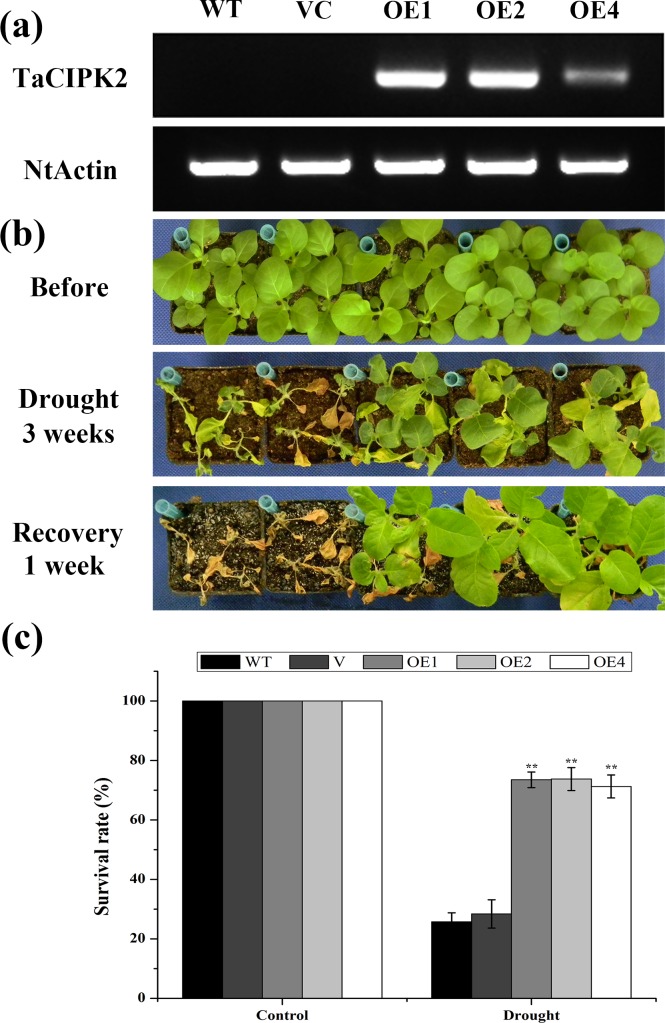
*TaCIPK2*-overexpressing lines have enhanced tolerance to drought stress in tobacco. (a) Expression level of *TaCIPK2* in the transgenic tobacco. (b) Phenotypes of WT, VC and *TaCIPK2*-overexpressing lines after withholding water for 3 weeks and re-watering for one week. (c) The survival rate of control and transgenic lines under normal and drought stress conditions. Asterisks indicate statistically significant differences (**P < 0.*01*) compared to the control lines. Error bars show the means ±SD calculated from three replicates.

The up-regulation of *TaCIPK2* expression in response to PEG, ABA, and H_2_O_2_ suggests that TaCIPK2 may have important functions in abiotic stress responses. To evaluate the impact of *TaCIPK2* over-expression on the drought tolerance of plants, transgenic tobacco plants overexpressing *TaCIPK2* were generated. Three independent transgenic T_2_ lines (OE1, OE2, and OE4) with different expression levels were chosen for further phenotypic assays (Fig 6A). Five-week-old controls (WT and VC) and *TaCIPK2* overexpressing lines were exposed to drought conditions (water was withheld for 3 weeks). All the three transgenic lines grew better than the control plants (Fig 6B) under treatment. After re-watering for 1 week, the survival rates of the control plants were considerably lower than for the *TaCIPK2*-overexpressing lines (Fig 6C). These results indicated that *TaCIPK2* expression may improve the ability of the transgenic tobacco plants to tolerate drought conditions.

**Fig 7:**
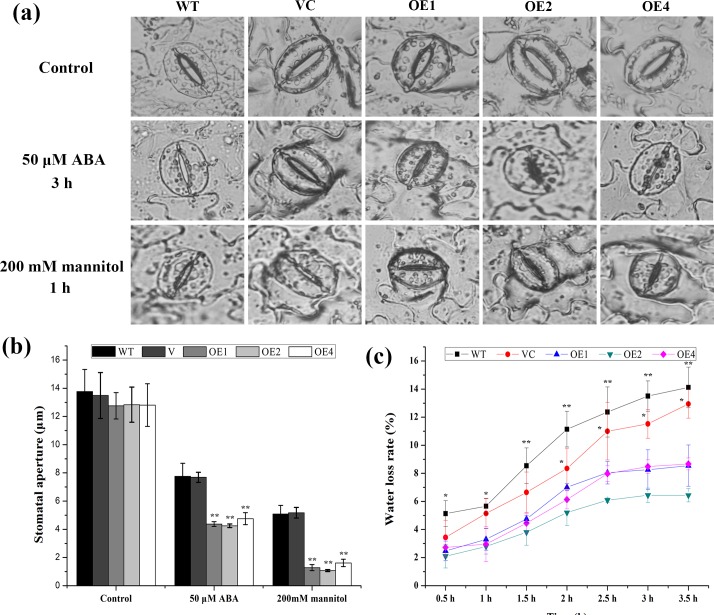
Water loss rate and stomatal movement of *TaCIPK2*-overexpressing transgenic lines under ABA and mannitol treatments. (a) Stomatal movement of 4-week-old WT, VC and *TaCIPK2*-overexpressing tobacco plants under 50 μM ABA and 200 mM mannitol treatments. Bright-field pictures were taken at 20x magnification using a fluorescence microscope. Scale bar = 100 μm. (b) Stomatal apertures were measured under normal conditions and 50 μM ABA and 200 mM mannitol treatments. The error bar was calculated based on the three independent replicate experiments. (c) Comparison of water loss rate between control and *TaCIPK2*-overexpressing lines. Three independent experiments were used to calculate the ±SD.

Drought tolerance is influenced by the abilities of plants to control their water content. Therefore, the rates of water loss were measured in *TaCIPK2*-overexpressing and control lines. Leaves were harvested and their fresh weight changes were recorded for every hour. As shown in Fig 7C, the three transgenic lines exhibited slower rates of water loss than the controls. These results indicated that the decreased rates of water loss contributed to the increased drought tolerance conferred by *TaCIPK2*-overexpression.

### *TaCIPK2* over-expression increased ABA-induced stomatal closure in transgenic plants

Stomata are important sites of water loss due to transpiration. Moreover, ABA induces stomatal closure under drought stress conditions. To investigate whether guard cells were more sensitive to ABA in *TaCIPK2*-overexpressing lines than the control, we examined the stomatal aperture status of the leaf abaxial epidermis. There was no difference in stomatal density in either the controls or the transgenic lines (S3 Fig). The stomatal apertures of the three *TaCIPK2*-overexpressing lines were smaller than in the control lines following treatment with 50 μM ABA for 3 h (Fig 7A). These results suggest that TaCIPK2 exhibits ABA sensitivity and influences ABA-regulated stomatal closure in the transgenic tobacco plants.

We also investigated the stomatal movement in the leaves of *TaCIPK2*-overexpressing tobacco plants grown under osmotic stress conditions. The stomatal apertures of the three transgenic lines were almost completely closed, while the stomatal apertures of the WT and VC lines remained slightly open after treatment with 200 mM mannitol for 1 h (Fig 7A and 7B). These results indicate that TaCIPK2 affects the stress responses in transgenic tobacco plants through regulating stomatal closure.

### Over-expression of *TaCIPK2* increases ABA sensitivity in transgenic plants

**Fig 8:**
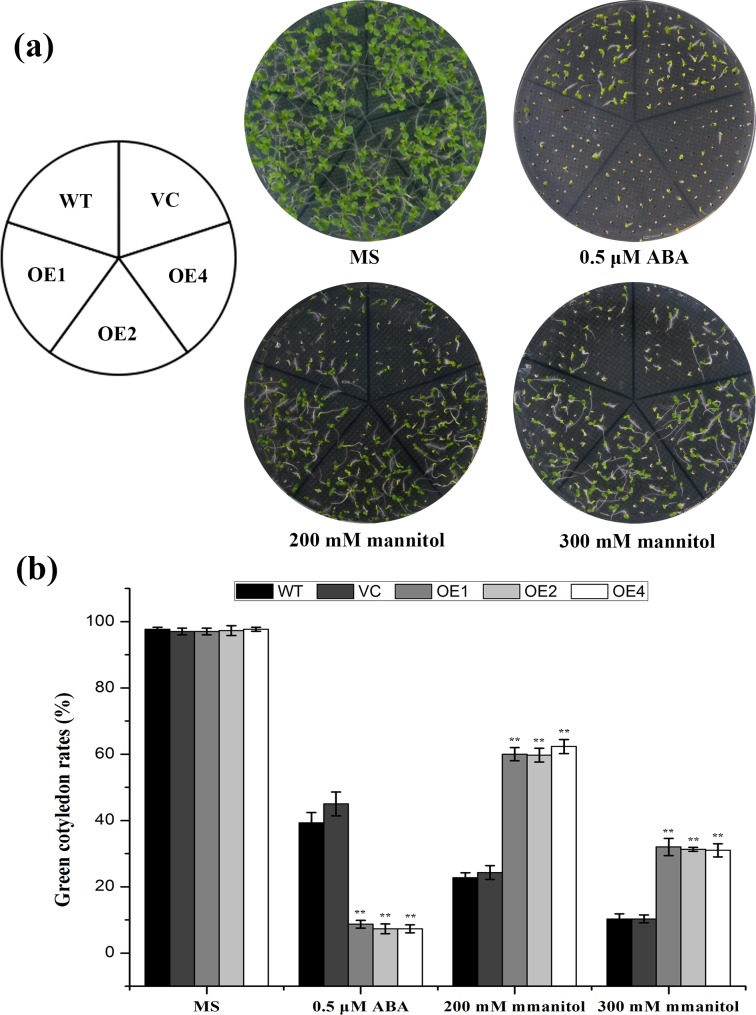
The analysis of green cotyledon rates in the controls (VC and WT) and *TaCIPK2*-overexpressing lines at early developmental stages. (a) The controls and the T_3_ generation of *TaCIPK2*-overexpressing tobacco seeds were germinated on MS media with or without 0.5 μM ABA, 200 mM mannitol and 300 mM mannitol and photographed after two weeks. (b) The green cotyledon rates were counted after germination. Vertical bars indicate ± SD calculated from three independent biological replicates with similar results.

In addition to phenotypic analysis, the drought stress tolerance of plants overexpressing *TaCIPK2* was further assessed by records of the germination rates. There was no significant difference in the germination rates of all lines on MS medium. However, the germination rates of the three transgenic lines were higher than for the control lines on selective media containing 200 or 300 mM mannitol (Fig 8A). These results confirmed that *TaCIPK2*-expressing transgenic tobacco plants were more drought tolerant than the WT and VC controls.

On the basal MS medium without ABA, three *TaCIPK2*-overexpressing lines (OE1, OE2, and OE4) had germination rates similar to the control lines, while the seeds germination rates of OE1, OE2, and OE4 were more affected by 0.5 μM ABA than the WT and VC seeds (Fig 8A). The rates of green cotyledon in the transgenic lines were 8.7%, 7.4%, and 7.3%, whereas of the corresponding rates in the WT and VC controls were 39.3% and 45.0% (Fig 8B). These observations indicated that *TaCIPK2*-overexpressing plants exhibited ABA hypersensitivity during the germination process.

### Variations in MDA and H_2_O_2_ contents, IL levels, and antioxidant enzyme activities under drought conditions

**Fig 9:**
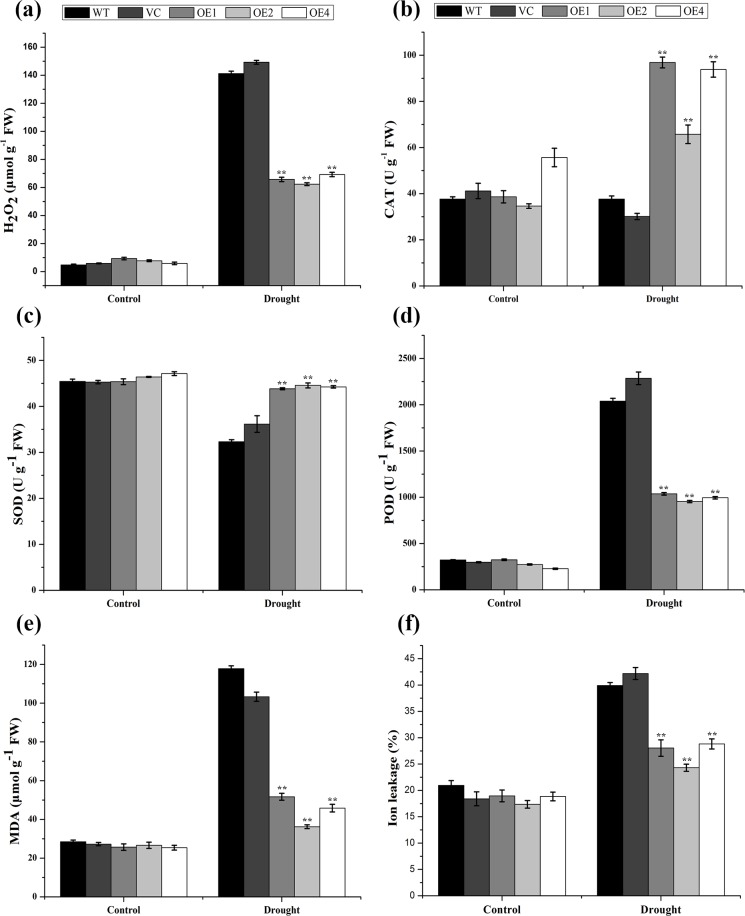
Physiological indices of control and *TaCIPK2*-overexpressing transgenic plants under drought stress conditions. Analysis of H_2_O_2_ content (a), CAT (b), SOD (c), and POD (d) activities, MDA content (e) and ion leakage (f) in control and *TaCIPK2*-overexpressing (OE) lines under normal and drought stress conditions. Values shown are means ± SE of three replicates. Asterisks indicate statistically significant differences from control (**P < 0.*01*).

The MDA content and IL level were used to represent the physiological status of the *TaCIPK2*-overexpressing transgenic tobacco plants following exposure to drought stress conditions. No difference was observed between the control and transgenic lines under normal conditions. However, under drought conditions, the MDA content and IL level were lower in the three transgenic lines than in the WT and VC control lines, indicating that there was less damage to the membranes of the transgenic plants (Fig 9E and 9F). These results confirmed that the *TaCIPK2*-overexpressing lines were more tolerant to drought stress conditions than the controls.

Applied stress damages plant cells and induces the production of reactive oxygen species (ROS), such as H_2_O_2_. Therefore, H_2_O_2_ was used to represent ROS levels. Additionally, relative activities of CAT, SOD and POD were used to indicate the ROS scavenging abilities of the control and *TaCIPK2*-overexpressing lines. The results showed that the H_2_O_2_ contents were similar among all lines under normal conditions, while the *TaCIPK2*-overexpression lines accumulated less amounts of H_2_O_2_ than the control plants following drought treatment (Fig 9A). Additionally, the activities of CAT and SOD were much higher in the *TaCIPK2*-overexpressing lines than in the control plants after exposure to drought stress; conversely, the POD activity levels were lower in *TaCIPK2*-overexpressing lines than in the control plants (Fig 9B–9D). The transgenic plants exhibited greater ROS scavenging abilities because of their increased CAT and maintained SOD activities. These results suggested that *TaCIPK2*-overexpressing lines exhibited increased drought stress tolerance due to enhanced ROS scavenging.

### TaCIPK2 regulates the expression of stress-responsive genes under drought stress conditions

**Fig 10:**
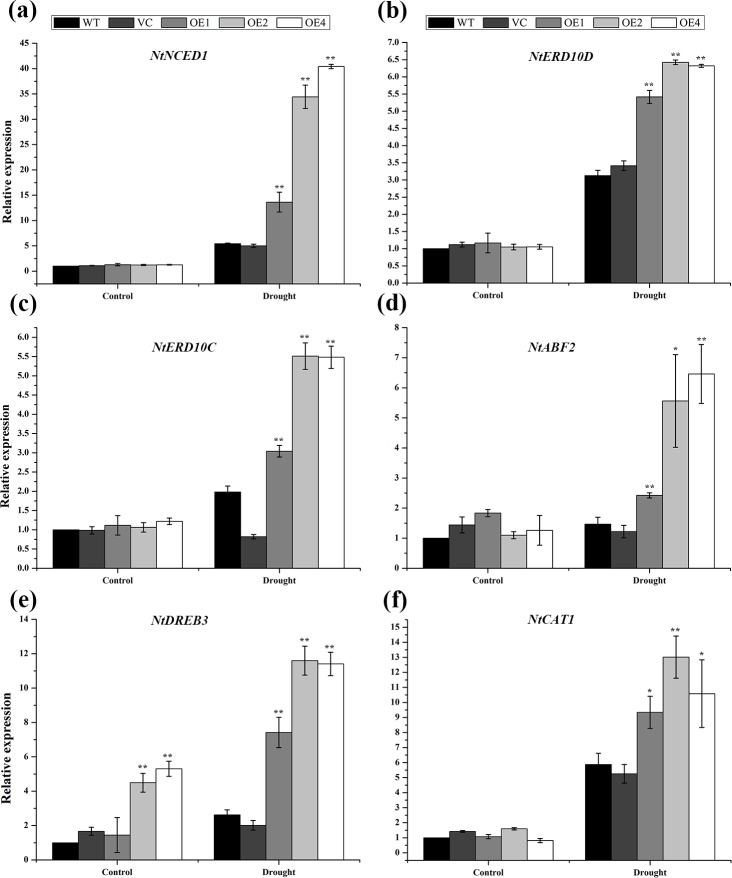
Expression patterns of relevant genes in control and *TaCIPK2*-overexpressing plants. Seedlings of WT and OE were subjected to drought for two weeks, and then total RNAs were extracted from the leaves. Related genes including (a) *NtNCED1*, (b) *NtERD10D*, (c) *NtERD10C*, (d) *NtABF2*, (e) *NtCAT1* and (f) *NtDREB3* were analyzed by qRT-PCR. Values are means ± SE of three replicates. Asterisks indicate statistically significant differences from WT (*P < 0.*05*; **P < 0.*01*) under drought stress.

To further characterize the molecular mechanism of TaCIPK2, the related genes including the ROS detoxification gene (*NtCAT1*), the dehydration-responsive element-binding gene (*NtDREB3*), abscisic acid related genes (*NtABF2* and *NtNCED1*) and stress response genes (*NtERD10C* and *NtERD10D*) were analyzed using the control plants and three *TaCIPK2*-overexpressing lines. Under drought conditions, all genes were significantly up-regulated in the *TaCIPK2*-overexpressing lines compared to the expression levels in the WT controls (Fig 10). These results illustrated that the over-expression of *TaCIPK2* in plants increases drought tolerance by inducing the expression of stress-responsive and ROS-related genes.

## Discussion

Many CIPKs participate in various stress-related responses in plants. Though more than 29 *CIPK* genes have been found in wheat, only a few of them had been functionally analyzed, such as *TaCIPK14* [15], *TaCIPK19* (*WPK* 4) [28], *TaCIPK24* [7] and *TaCIPK29* [29]. In this study, we found that *TaCIPK2* was up-regulated in wheat leaves by PEG, H_2_O_2_, and ABA treatments (Fig 3B–3D), implying a potential role of TaCIPK2 in stress responses. *TaCIPK2*-overexpressing tobacco plants exhibited improved drought tolerance (Fig 6B), which is consistent with the effects of other previously studied CIPKs. For example, ZmCIPK8 enhances the drought tolerance of transgenic tobacco seedlings by regulating stress-related genes [30]. The over-expression of *BrCIPK1* conferred drought tolerance in rice, which was associated with increasing proline content [31]. Rice *cipk31* mutants were reported to exhibit retarded germination and delayed seedling growth [32].

Previous studies indicated that CBL proteins could specifically interact with relevant CIPKs [33–35] and that it was essential for the activation of CIPKs to interact with CBLs [36]. Thus we examined the interaction between TaCIPK2 and TaCBLs through the yeast two-hybrid method. The results showed that four TaCBL proteins could interact with TaCIPK2 in vitro, illustrating that a CIPK protein could interact with one or more CBL proteins.

Drought stress is among the most important factors influencing plant growth and development. Plants have developed a series of mechanisms to cope with serious water shortages. Among these mechanisms, the ABA-mediated control of stomatal apertures is important for reducing water loss. In this study, transgenic tobacco plants exhibited greater stomatal closure and lower water loss rate than control plants following ABA and mannitol treatments (Fig 7A and 7C). Furthermore, ABA-regulated S-type anion channel activation is a crucial for increasing cytosolic free Ca^2+^ content, which initiates certain downstream pathways [35, 37, 38]. The CBL/CIPK complex is a crucial Ca^2+^ signal transduction component, and may have latent roles in ABA-regulated stomatal movement. Therefore, the smaller stomatal aperture was likely responsible for the increased drought tolerance of the transgenic tobacco lines.

Several stresses, including drought, can lead to the over-accumulation of cell-damaging ROS species. The MDA, H_2_O_2_, and IL levels are usually used to represent ROS-caused membrane damage [39, 40]. Our findings revealed that the MDA, H_2_O_2_, and IL levels in the leaves of transgenic plants over-expressing *TaCIPK2* were lower than in control leaves under drought condition (Fig 9A, 9E and 9F). This result suggests that the improved drought resistance of the *TaCIPK2*-overexpressing plants may be partly attributed to enhanced cell membrane stability. Additionally, antioxidant enzymes activities (CAT and SOD) in transgenic plants were higher than in the control plants after drought treatment (Fig 9B and 9C). These results indicate that TaCIPK2 confers drought tolerance by mediating the ROS scavenging system.

For further analysis of the molecular mechanisms in *TaCIPK2*-overexpressing plants under drought stresses, the expression levels of six related marker genes (*NtCAT1*, *NtDREB3*, *NtABF2*, *NtNCED1*, *NtERD10C* and *NtERD10D*) were detected. The results showed that the expression levels of the antioxidant enzyme (*NtCAT*) were higher in *TaCIPK2*-overexpressing lines under stress treatments (Fig 10F), revealing that *TaCIPK2*-overexpressing plants had higher CAT enzyme activities and lower H_2_O_2_ contents under drought conditions (Fig 9A and 9B). In addition, the expression of *DREB3* and *ABF2* could be induced by dehydration conditions [41], and the expression levels of the two genes showed higher induction under drought treatments (Fig 10A and 10D). Previous studies showed that *NtNECD1* played an essential role in regulating ABA biosynthesis [42], while the LEA protein family members (*NtERD10C* and *NtERD10D*) could stabilize cellular structures during stress treatments [43, 44]. The expression levels of the above mentioned genes were up-regulated in *TaCIPK2*-overexpressing plants under drought treatments (Fig 10). Therefore, the over-expression of *TaCIPK2* in tobacco plants could improve drought tolerance by increasing the expression levels of stress-responsive and ROS-related genes.

In conclusion, TaCIPK2 is a stress-responsive protein kinase that confers drought tolerance in plants by enhancing cell membrane stability and regulating the ROS scavenging system. TaCIPK2 also contributes to drought tolerance by mediating ABA-induced stomatal closure. Future research should focus on the stomatal movement mechanism regulated by TaCIPK2, which will increase our understanding of how TaCIPK functions in abiotic stress responses.

## Supporting Information

S1 Fig The subcellular localization of TaCIPK2 was on the onion epidermal cells. The fusion protein CIPK2-GFP was transiently expressed in onion epidermal cells and the tissue was treated with 30% sucrose solution. Scale bar = 200 μm. The green fluorescence signals were observed by fluorescence microscopy (LX71, Olympus, Japan).
(TIF)

S2 Fig The predicted myristoylation and palmitoylation sites of TaCIPK2. The G2 and G6 were myristoylation sites, and the C300 showed palmitoylation site.
(TIF)

S3 Fig Stomatal density of the tobacco epidermal cells in the control and *TaCIPK2*-overexpressing plants. (TIF)

S1 Table Primers used for PCR analysis. (DOC)

## Abbreviations

CIPK: CBL -interacting protein kinases
CBLs: calcineurin B-like proteins
PEG: polyethylene glycol
ABA: abscisic acid
CaMV: Cauliflower Mosaic Virus
MS: Murashige and Skoog
CAT: Catalase
IL: Ion leakage
MDA: Malonaldehyde
OE: Overexpression
POD: Peroxidase
qRT-PCR: Real-time quantitative PCR
ROS: Reactive oxygen species
RT-PCR: Reverse transcription–PCR
SOD: Superoxide dismutase
VC: Vector control
WT: Wild type
