# Supplementary figures and images for: A CBL-Interacting Protein Kinase TaCIPK2 Confers Drought Tolerance in Transgenic Tobacco Plants through Regulating the Stomatal Movement
Source: PLoS One. 2016 Dec 9;11(12):e0167962. doi: 10.1371/journal.pone.0167962 (PMC5148042; doi:10.1371/journal.pone.0167962)

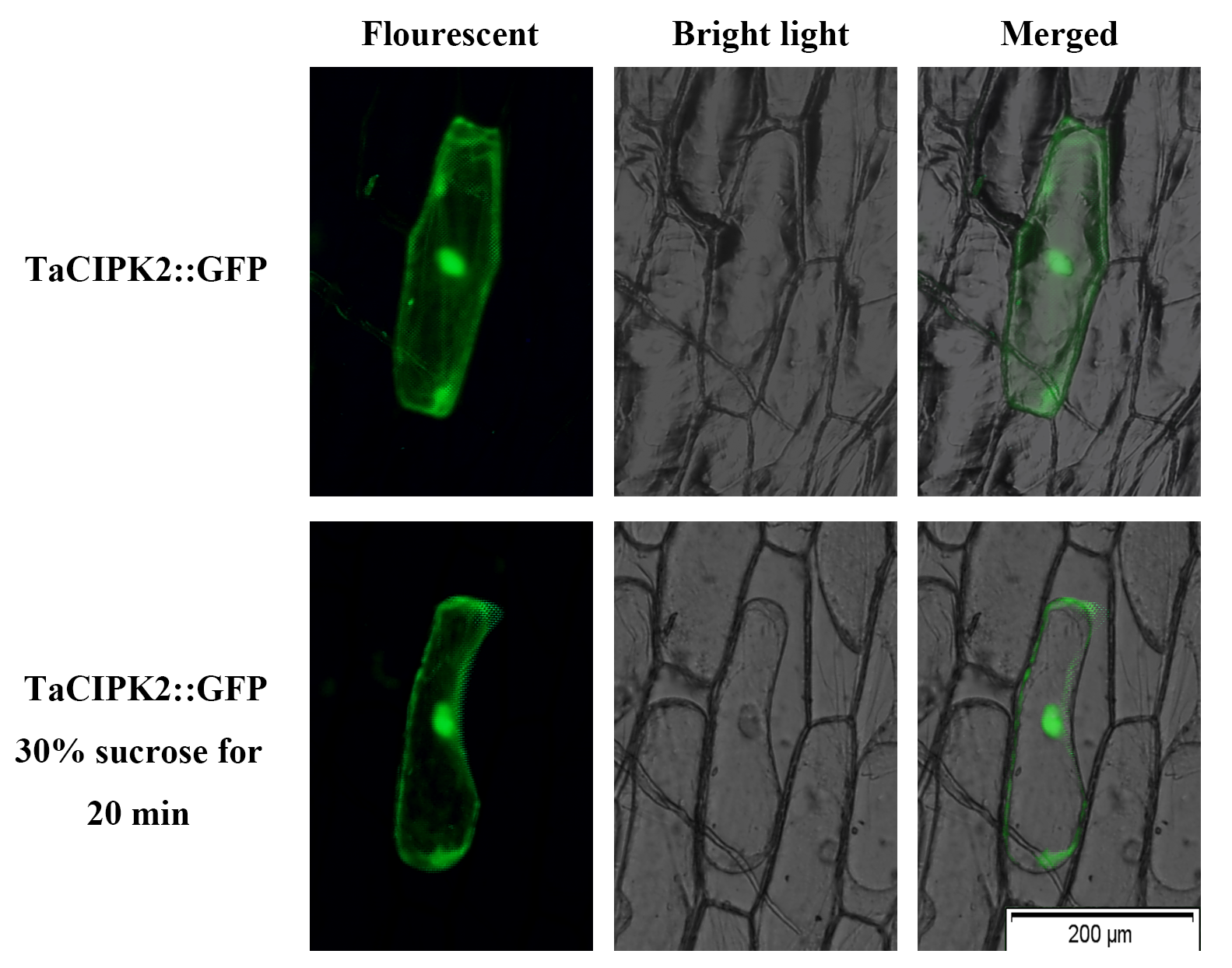

Supplement: S1 Fig — The fusion protein CIPK2-GFP was transiently expressed in onion epidermal cells and the tissue was treated with 30% sucrose solution. Scale bar = 200 μm. The green fluorescence signals were observed by fluorescence microscopy (LX71, Olympus, Japan). (TIF) [file pone.0167962.s001.tif]

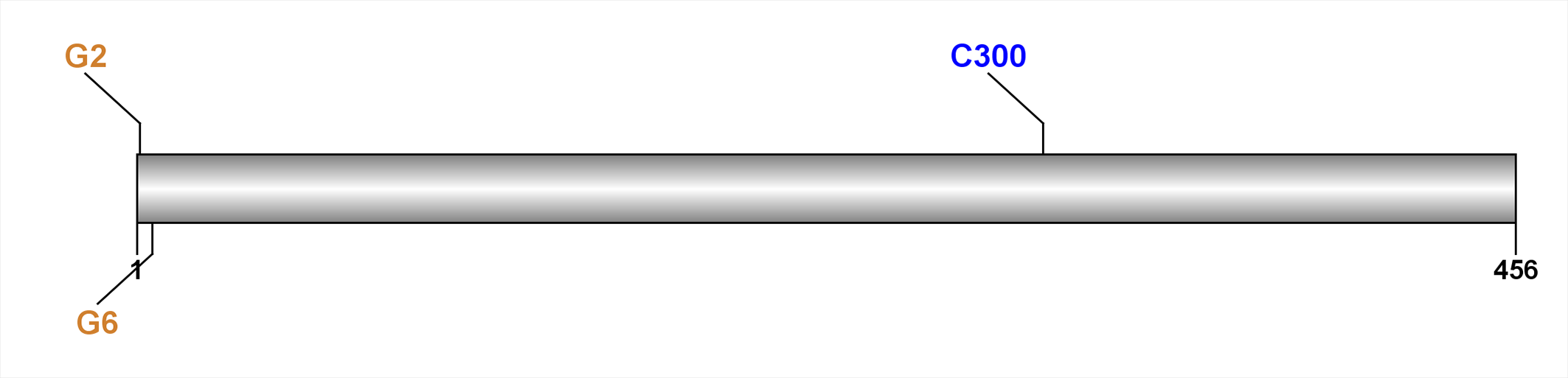

Supplement: S2 Fig — The G2 and G6 were myristoylation sites, and the C300 showed palmitoylation site. (TIF) [file pone.0167962.s002.tif]

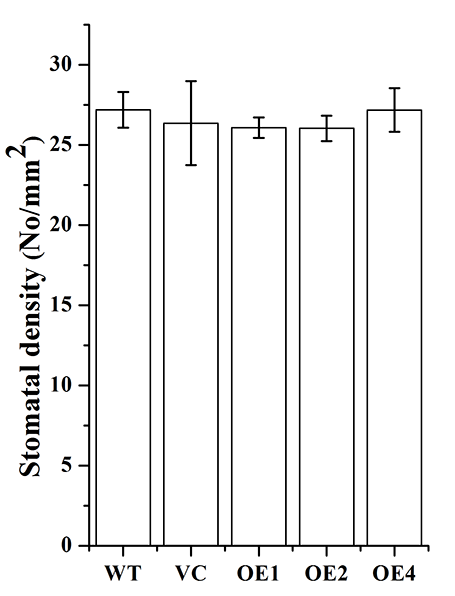

Supplement: S3 Fig — (TIF) [file pone.0167962.s003.tif]
