# Supplementary material for: A CBL-Interacting Protein Kinase TaCIPK2 Confers Drought Tolerance in Transgenic Tobacco Plants through Regulating the Stomatal Movement
Source: PLoS One. 2016 Dec 9;11(12):e0167962. doi: 10.1371/journal.pone.0167962 (PMC5148042; doi:10.1371/journal.pone.0167962)
Supplement: S1 Table — (DOC) [file pone.0167962.s004.doc]

**S1 Table. Primers used for PCR analysis.**

| Name | Oligonucleotides (5′~3′) |
| --- | --- |
| *TaCIPK2RT-F* | GCGCTCAAGGACATAGTCTGG |
| *TaCIPK2RT-R* | TTGCACCTGGCACAGTTC |
| *GFPTaCIPK2-F* | TCTAGAATGGGAGAGCAGAAGGGGAA |
| *GFPTaCIPK2-R* | GGGCCCACATGGTTGCTGCTGTGGC |
| *OETaCIPK2-R* | GGGCCCTTAACATGGTTGCTGCTGTGGC |
| *ADTaCIPK2-F* | GGAATTCCATATGGGAGAGCAGAAGGGGAA |
| *ADTaCIPK2-R* | CCGGAATTCACATGGTTGCTGCTGTGGC |
| *TaActin-F* | TCTATTTTGGCCTCTCTTAGCAC |
| *TaActin-R* | TTTCCTGTACCCCTTATTCCTC |
| *NtACT2A-F* | CTATTCTCCGCTTTGGACTTGGCA |
| *NtACT2B-R* | ACCTGCTGGAAGGTGCTGAGGGAA |
| *NtDREB3-F* | GCCGGAATACACAGGAGAAG |
| *NtDREB3-R* | CCAATTTGGGAACACTGAGG |
| *NtCAT -F* | AGGTACCGCTCATTCACACC |
| *NtCAT-R* | AAGCAAGCTTTTGACCCAGA |
| *NtERD10D-F* | GAGGACACGGCTGTACCAGT |
| *NtERD10D-R* | GCGCCACTTCCTCTGTCTT |
| *NtERD10C-F* | AACGTGGAGGCTACAGATCG |
| *NtERD10C-R* | GTTCCTCTTGGGCATGAGTT |
| *NtNCED1-F* | AAGAATGGCTCCGCAAGTTA |
| *NtNCED1-R* | GCCTAGCAATTCCAGAGTGG |
| *NtABF2-F* | GCAGCCATCTATCTATTC |
| *NtABF2-R* | GCAACTCATCCATATTCA |
| *pGFP-R* | GCATGGCGCTCTTGAAGAAGT |
